# Effectiveness of Golimumab as Second Anti-TNFα Drug in Patients with Rheumatoid Arthritis, Psoriatic Arthritis and Axial Spondyloarthritis in Italy: GO-BEYOND, a Prospective Real-World Observational Study

**DOI:** 10.3390/jcm11144178

**Published:** 2022-07-19

**Authors:** Salvatore D’Angelo, Enrico Tirri, Angela Maria Giardino, Marco Mattucci-Cerinic, Lorenzo Dagna, Leonardo Santo, Francesco Ciccia, Bruno Frediani, Marcello Govoni, Francesca Bobbio Pallavicini, Rosa Daniela Grembiale, Andrea Delle Sedie, Rita Mulè, Francesco Paolo Cantatore, Rosario Foti, Elisa Gremese, Paola Conigliaro, Fausto Salaffi, Ombretta Viapiana, Alberto Cauli, Roberto Giacomelli, Luisa Arcarese, Giuliana Guggino, Romualdo Russo, Amy Puenpatom, Domenico Capocotta, Francesca Nacci, Maria Grazia Anelli, Valentina Picerno, Corrado Binetti, Florenzo Iannone

**Affiliations:** 1 Istituto Reumatologico Lucano (I.Re.L), Dipartimento Regionale di Reumatologia, AOR San Carlo di Potenza, 85100 Potenza, Italy; valepicerno@tiscali.it; 2 UOSD di Reumatologia, Ospedale San Giovanni Bosco, 80144 Napoli, Italy; entirri@tin.it (E.T.); dome.capocotta@hotmail.it (D.C.); 3 Medical Affairs MSD Italia S.r.l., 00189 Rome, Italy; angela.giardino@msd.com (A.M.G.); corrado.binetti@msd.com (C.B.); 4 A.O. Careggi Università, 50134 Firenze, Italy; mmcerinic@gmail.com; 5 Unit of Immunology, Rheumatology, Allergy and Rare Diseases (UnIRAR), IRCCS San Raffaele Scientific Institute, Vita-Salute San Raffaele University, 20132 Milan, Italy; dagna.lorenzo@unisr.it; 6 U.O.S. Reumatologia ASL BT—DSS 4, 76121 Barletta, Italy; leonardo.santo1@tin.it; 7 AOU Università degli Studi della Campania “Luigi Vanvitelli”, 80138 Napoli, Italy; francesco.ciccia@unicampania.it; 8 UOC di Reumatologia, Azienda Ospedaliero Universitaria Senese, 53100 Siena, Italy; fredianibruno60@gmail.com; 9 AOU S. Anna di Ferrara, UOC Reumatologia, Dipartimento di Scienze Mediche, Università di Ferrara, 44121 Ferrara, Italy; gvl@unife.it; 10 Fondazione IRCCS Policlinico San Matteo S.C. Reumatologia, 27100 Pavia, Italy; f.bobbiopallavicini@smatteo.pv.it; 11 Rheumatology Research Unit, Dipartimento di Scienze della Salute, Università degli studi “Magna Graecia” di Catanzaro, 88100 Catanzaro, Italy; rdgrembiale@unicz.it; 12 U.O. Reumatologia Azienda Ospedaliero Universitaria Pisana, 56126 Pisa, Italy; adellesedie@gmail.com; 13 IRCCS Azienda Ospedaliero-Universitaria di Bologna, Policlinico di Sant’Orsola, UO Reumatologia, 40138 Bologna, Italy; rita.mule@aosp.bo.it; 14 UOC Reumatologia Universitaria, “Ospedali Riuniti” di Foggia, 71122 Foggia, Italy; francescopaolo.cantatore@unifg.it; 15 A.O.U. Policlinico G. Rodolico-S. Marco, U.O di Reumatologia, 95123 Catania, Italy; rosfoti5@gmail.com; 16 Fondazione Policlinico Universitario A. Gemelli-IRCCS, Università Cattolica del Sacro Cuore, 00168 Rome, Italy; elisa.gremese@unicatt.it; 17 UOC Reumatologia, Dipartimento di Medicina dei Sistemi, Università di Roma “Tor Vergata”, 00133 Rome, Italy; paola.conigliaro@uniroma2.it; 18 Clinica Reumatologica, Ospedale “C. Urbani” Università Politecnica delle Marche, 60035 Ancona, Italy; fausto.salaffi@gmail.com; 19 Ospedale Borgo Roma Policlinico G.B. Rossi, U.O.C. Reumatologia, 37126 Verona, Italy; ombretta.viapiana@univr.it; 20 Azienda Osped/Universitaria Policlinico Monserrato, 09124 Cagliari, Italy; cauli@unica.it; 21 UOC ImmunoReumatologia, Università CampusBio Medico di Roma, 00128 Rome, Italy; r.giacomelli@unicampus.it (R.G.); l.arcarese@unicampus.it (L.A.); 22 PROMISE Department, University of Palermo, 90133 Palermo, Italy; giuliana.guggino@unipa.it; 23 AORN, A. Cardarelli U.O.S. Reumatologia, 80131 Napoli, Italy; romualdor90@libero.it; 24 Center for Observational and Real-World Evidence, Merck & Co., Inc., Kenilworth, NJ 07033, USA; puenpatom.amy@merck.com; 25 Department of Experimental and Clinical Medicine, Divisions of Rheumatology AOUC, University of Florence, 50134 Florence, Italy; clintrial@icloud.com; 26 Rheumatology Unit, DETO, University of Bari, 70124 Bari, Italy; marynelli@libero.it (M.G.A.); florenzo.iannone@uniba.it (F.I.)

**Keywords:** golimumab, anti-TNF inhibitor, biologic, rheumatoid arthritis, psoriatic arthritis, axial spondyloarthritis

## Abstract

In this prospective observational study, data were collected from 34 rheumatology clinics in Italy in patients with rheumatoid arthritis (RA), psoriatic arthritis (PsA) and axial spondyloarthritis (axSpA) who started golimumab (GLM) as a second anti-TNFα drug. The primary objective was to evaluate the effectiveness of GLM after 6 months. Changes in quality of life using the EQ-5D-5L were also assessed. A total of 194 patients aged 53.2 ± 12 years started GLM as a second anti-TNF drug: 39 (20.1%) with RA, 91 (46.9%) with PsA and 64 (32.9%) with axSpA. After 6 months of GLM treatment, 68% of RA patients achieved low disease activity (LDA; DAS28-CRP ≤ 3.2), 31.9% of PsA patients achieved minimal disease activity and 32.5% of axSpA patients achieved LDA (ASDAS-CRP < 2.1). Good/moderate EULAR response was achieved in 61.9% and 73.8% of patients with RA and PsA, respectively, and 16% of axSpA patients achieved a 50% improvement in BASDAI. Across all indications, improvements in disease activity measures and EQ-5D-5L domains were observed over 6 months. The main reasons for GLM interruption were lack/loss of efficacy (7.2%) or adverse events (2%). This study confirms the effectiveness of GLM as a second-line anti-TNF for the treatment of RA, PsA and axSpA in a real-world setting in Italy.

## 1. Introduction

Following the availability of antibodies targeting tumor necrosis factor-alpha (TNFα), the standard of therapeutic care offered to patients with rheumatoid arthritis (RA), psoriatic arthritis (PsA) and spondyloarthritis (SpA) has substantially improved in terms of controlling signs/symptoms of inflammation, quality of life and functional outcome [1,2,3,4].

Whilst anti-TNF drugs are the most frequently used first-line bDMARDs, the response can decline over time in some patients, necessitating the switch to another biologic [5,6,7,8,9,10]. Data from real-life settings show that as many as 40–50% of patients stop their first anti-TNF treatment due to loss of therapeutic effectiveness; this is typically seen after a good initial response or due to adverse events (AEs) [8,9].

Recent guidelines for the management of RA, PsA and axial SpA (axSpA) have acknowledged the need for switching due to first-line failure and have been updated accordingly. In patients with RA, the European Alliance of Associations for Rheumatology (EULAR) guidelines recommend that if one anti-TNF therapy fails, patients may receive another anti-TNF agent or another biologic with a different mode of action [10]. Similarly, in patients with axSpA, EULAR guidelines recommend switching to another anti-TNF drug or an anti-IL-17 agent if anti-TNF treatment fails [5]. In patients with PsA who fail to respond to a biological disease-modifying antirheumatic drug (bDMARD), it is recommended to switch to another anti-TNF therapy or another bDMARD [6].

Among the other four licensed anti-TNF inhibitors approved for the treatment of RA, PsA and axSpA (adalimumab, infliximab, etanercept and certolizumab pegol), golimumab (GLM) has also been shown to be effective in RA [11], PsA [12], ankylosing spondylitis (AS) [13], and non-radiographic axSpA [14,15] from randomized clinical trials (RCTs). GLM also has the longest dosing intervals (once monthly) among subcutaneous anti-TNF drugs. Besides improving signs and symptoms in these patients, a high rate of retention on GLM is achieved in biological-naïve patients, with about 70% maintaining treatment through 5 years [16].

The efficacy of GLM after failure with other anti-TNF drugs was evaluated in the prospective GO-AFTER study [17]. GLM was shown to be effective and safe for patients with RA who had failed one or more anti-TNF drugs, with results confirmed through 5 years in the long-term extension study [18].

However, in patients with PsA or axSpA, data on the effectiveness of GLM following the first-line anti-TNF failure are scarce [19,20]. To address this gap, recent real-life retrospective studies performed in Spain [21], Turkey [22], France [23,24], Germany [25] and Italy [26] have examined the long-term effectiveness and retention rate of GLM. The present prospective study, GO-BEYOND, was designed to evaluate the effectiveness of GLM as a second anti-TNF drug in patients with RA, axSpA or PsA who discontinued a first anti-TNF drug.

## 2. Patients and Methods

### 2.1. Patients and Study Design

GO-BEYOND was a prospective observational study including patients with a diagnosis, by the treating rheumatologists, of RA, PsA or axSpA. Patients were enrolled between July 2017 and December 2019, with visits at baseline, 3 and 6 months. The main analysis for this manuscript evaluated the effectiveness after 6 months of GLM therapy. All patients fulfilled 2010 classification criteria for RA [27], or CASPAR criteria for PsA [28] or ASAS criteria for axial (radiographic and non-radiographic) SpA [29]. Patients were treated across 34 Italian Rheumatology Centers with GLM after first-line anti-TNFα inhibitor failure. Inclusion criteria were: male or female patients ≥18 years diagnosed with active RA, PsA or axSpA, eligible for treatment with GLM according to the SmPC [30]; patients previously treated with an anti-TNFα inhibitor with or without methotrexate (MTX) for at least 6 months and experiencing anti-TNFα treatment failure due to loss of efficacy; patients who stopped an effective initial anti-TNFα treatment for at least 3 months due to discomfort or tolerability issues after taking treatment; agreement from female subjects to use adequate contraception to prevent pregnancy. The following contraindications to GLM according to the Summary of product characteristics (SmPC) [30] prevented patients from being included in the study: moderate or severe heart failure according to New York Heart Association Class III/IV; tuberculosis or other severe infections such as sepsis, abscesses and other opportunistic infections; hypersensitivity or clinically serious adverse drug reaction to the active substance or to any of the excipients. Other exclusion criteria included: current or previous cancer in the past 5 years and current participation in an interventional trial or another non-interventional study, excluding registries. The study (protocol number: MK-8259-6415) was approved by the Regional Ethics Committee for Clinical Studies from the Tuscany Region (Sezione, Area Vasta Sud Est) on 20 March 2017. Local ethics committee approval from all participating centers and written informed consent for the anonymous use of personal data were also obtained from every patient, in compliance with Legislative Decree 196/2003. This study complies with the ethical standards laid down in the 1975 Declaration of Helsinki.

The GO-BEYOND database recorded demographic features at the baseline visit (age, sex, and time since RA, PsA or axSpA diagnosis) and at each visit (visits 0, 3, and 6 months) the following measures: Disease Activity Score in 28 joints (DAS28) [31] for patients with RA and PsA, Ankylosing Spondylitis Disease Activity Score based on CRP (ASDAS-CRP) [32,33], Bath Ankylosing Spondylitis Disease Activity Index (BASDAI) [34], the Assessment of SpondyloArthritis international society Health Index (ASAS HI) [35] for patients with axSpA and health-related quality of life (HRQoL) assessed using the EuroQoL 5-Dimension 5-Level (EQ-5D-5L) [36] for all three patient groups. For peripheral joint assessment, 68 joints were assessed for tenderness, and 66 joints were assessed for swelling (for patients with RA and PsA). Rheumatoid factor (RF), anti-citrullinated protein antibody (ACPA) (for patients with RA) and human leukocyte antigen B27 (HLAB27) were also measured at baseline (for patients with axSpA).

### 2.2. Outcome Measures

The primary outcome of the present study was to assess the effectiveness of GLM treatment after 6 months. In patients with RA, this was defined as the proportion (%) of patients achieving low disease activity (LDA; DAS28-CRP ≤ 3.2) according to EULAR guidelines [37]. For patients with PsA, the proportion of patients achieving MDA was evaluated. The MDA criterion is a score of 7 outcome measures in PsA, and patients were classified with MDA when 5 out of 7 outcome measures were fulfilled as previously described [38]. The effectiveness of GLM in axSpA was defined as the proportion of patients achieving at least LDA according to ASDAS-CRP (<2.1) [32,33]. In patients with axSpA, we measured BASDAI 50, defined as a 50% improvement or more of the baseline BASDAI after treatment with GLM. The EULAR response criteria were used to evaluate the individual change in DAS28-CRP from baseline and the level of DAS28-CRP reached at 6 months in patients with RA and PsA classified as good responders, moderate responders or non-responders [39]. HRQoL was assessed using the EQ-5D-5L [36]. Discontinuation of treatment and causes thereof were also recorded over the 6-month treatment period.

### 2.3. Sample Size Calculation

Based on the prevalence of RA, PsA and axSpA and the propensity of switching between anti-TNFα therapy in clinical practice (higher for RA) [19,40], it was estimated to enroll approximately 100 RA patients, 85 PsA patients and 65 axSpA patients. However, a greater number of axSpA patients and a lower number of RA patients were observed in clinical practice after a review of the feasibility of enrolment in each study center. Considering a drop-out rate of 10% expected to be lost to follow-up, we estimated the final number of patients to be 200: 40 with RA, 85 with PsA and 75 with axSpA should have been enrolled in about 30 investigational sites treating rheumatologic diseases in Italy. Assuming a response rate of 30% for each disease and the aforementioned sample sizes, the width of the 95% confidence interval (CI) is 0.3 for RA, 0.19 for PsA and 0.21 for axSpA. Sample size calculations were performed using PASS software (NCSS, LLC, Kaysville, UT, USA).

### 2.4. Statistical Analysis

Continuous variables are presented as mean and standard deviation, median, 25th and 75th percentiles. Categorical variables were summarized using absolute and relative frequencies (percentages). Percentages were calculated together with the corresponding 95% confidence interval computed using Clopper–Pearson method [41]. Data were initially evaluated for normal distribution with the Shapiro–Wilk test, and then the differences in disease activity scores at 3 and 6 months compared to baseline values were assessed using the paired-samples *t*-test or the paired-samples Sign Test, as appropriate. Differences at baseline and 6 months in the proportion of patients with problems across the 5 domains of the EQ-5D-5L (mobility, self-care, usual activities, pain/discomfort and anxiety/depression) were tested using the McNemar Test. Missing data for the calculation of disease-specific measures (e.g., BASDAI score, ASAS HI score) were handled according to the recommendation given in the literature for the specific score. A *p*-value of <0.05 was considered statistically significant, and all analyses were performed using SPSS statistical software, version 23.0 (SPSS, Chicago, IL, USA). There were no changes in the planned analyses of the study due to the COVID-19 pandemic.

## 3. Results

### 3.1. Baseline Clinical Characteristics

In total, 194 patients were included in the GO-BEYOND study: 39 (20.1%) with RA, 91 (46.9%) with PsA and 64 (32.9%) with axSpA. The baseline clinical characteristics of the three patient groups are summarized in Table 1. The majority of patients were female (N = 110; 56.7%), with a higher proportion in patients with RA (N = 29; 74.4%) compared to PsA (N = 47: 51.6%) and axSpA (N = 34; 53.1%) groups. Mean age and disease duration were also slightly higher in patients with RA (55.4 ± 11.4 years and 11 ± 9.1 years, respectively) compared to PsA (53.7 ± 11.3 years and 9.8 ± 7.8 years, respectively) and axSpA groups (51 ± 13.2 years and 9.2 ± 8.5 years, respectively). Comorbidities were highly prevalent (RA 66.7%, PsA 65.9% and axSpA 76.6%); hypertension (30.9%), thyroid disease (13.9%) and dyslipidemia (13.4%), were the most frequent. All patients had moderate-to-active disease, as observed by baseline mean ESR and CRP levels (particularly for patients with RA with mean CRP of 10.2 ± 18.1 mg/L and ESR of 23.6 ± 22.1 mm/h), DAS28-CRP for patients with RA and PsA (4.1 ± 0.94 and 3.8 ± 0.99, respectively), ASDAS-CRP (2.9 ± 0.97) and BASDAI (6 ± 2.1) for axSpA patients. The majority (N = 142; 73.2%) of patients were receiving concomitant medication, and a higher proportion of RA patients were receiving MTX (94.9%) or corticosteroids (38.5%) compared to PsA or axSpA patients, who were receiving more NSAIDs (~20–30%) (Table 2).

### 3.2. Previous Anti-TNFα Therapy and Reasons for Switching to GLM

Previous anti-TNFα drugs were mainly etanercept (N = 86; 44.3%) and adalimumab (N = 82; 42.3%) followed by infliximab (N = 17; 8.8%) and certolizumab (N = 9; 4.6%) (Table 2). Mean duration of previous biologic treatment was 49.9 ± 45.2 months, with infliximab administered longer (76.9 ± 63.4 months) compared to the other anti-TNFα drugs (about 30–50 months). Etanercept was mainly used in patients with RA and PsA, while adalimumab was mainly used to treat axSpA. The reasons for switching from initial anti-TNFα to GLM therapy were loss of efficacy (N = 155; 79.9%), injection-site or infusion reactions (N = 11; 5.7%), lack of compliance (N = 4; 2.1%), patient dissatisfaction (e.g., about handling or application frequency) (N = 2; 1%) and other (unspecified) adverse events (AEs) (N = 22; 11.3%), (Table 3).

### 3.3. Effectiveness of Golimumab in Patients with RA

**Figure 1:**
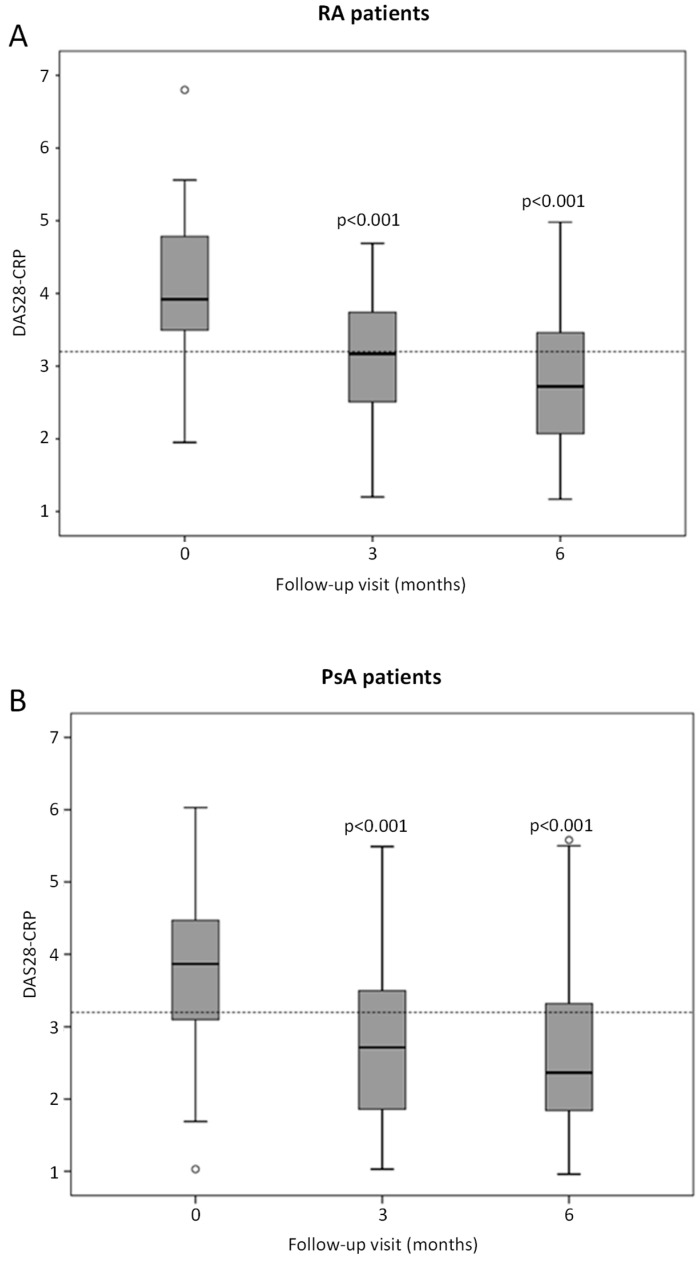
Box and whiskers plots illustrating changes in DAS28-CRP in RA and PsA patients at baseline, 3 and 6 months of treatment with GLM. (**A**) RA patients and (**B**) PsA patients. Data are presented as median, 25th/75th percentiles and maximum/minimum recorded values. Small open dots represent outliers, and horizontal dotted line represents a cut-off value of 3.2 (≤3.2 defined as low disease activity according to DAS28-CRP). Statistically significant differences compared to baseline values are represented by *p*-values.

After 6 months of GLM treatment for RA, 68% (95% CI: 46.5–85.1%) of patients achieved at least LDA, with 40% (95% CI: 21.1–61.3%) achieving remission, based on DAS28-CRP, while a good/moderate EULAR response was seen in 61.9% (95% CI: 38.4–81.9%). At baseline, the median DAS28-CRP score was 3.92 (interquartile range, IQR: 3.5–4.79) and decreased over time to 2.72 (IQR: 2.07–3.46; *p* < 0.001) at 6 months (Figure 1A). Mean SJC, TJC and PGA significantly decreased at 6 months compared to baseline values. CRP levels were also observed to decrease after 6 months, but this decrease did not reach statistical significance. ESR levels remained unchanged after 6 months compared to baseline values (Table 4).

### 3.4. Effectiveness of Golimumab in Patients with PsA

At 6 months, MDA was achieved in 31.9% (95% CI: 21.4–44.0%), and DAS28-CRP-based disease remission was achieved in 59.4% (95% CI: 46.4–71.5%) of patients with PsA. Furthermore, 73.8% (95% CI: 58.0–86.1%) of patients achieved a good/moderate EULAR response. DAS28-CRP score also decreased over the follow-up period (3.87, IQR: 3.1–4.47 at baseline to 2.37, IQR: 1.84–3.32 at 6 months; *p* < 0.001) (Figure 1B). Although a generalized decrease in mean CRP, ESR, SJC, TJC as well as PASI, VAS pain and PGA was observed at 6 months compared to baseline values, statistical significance was only detected for SJC, TJC and PGA (Table 4).

### 3.5. Effectiveness of Golimumab in Patients with AxSpA

**Figure 2:**
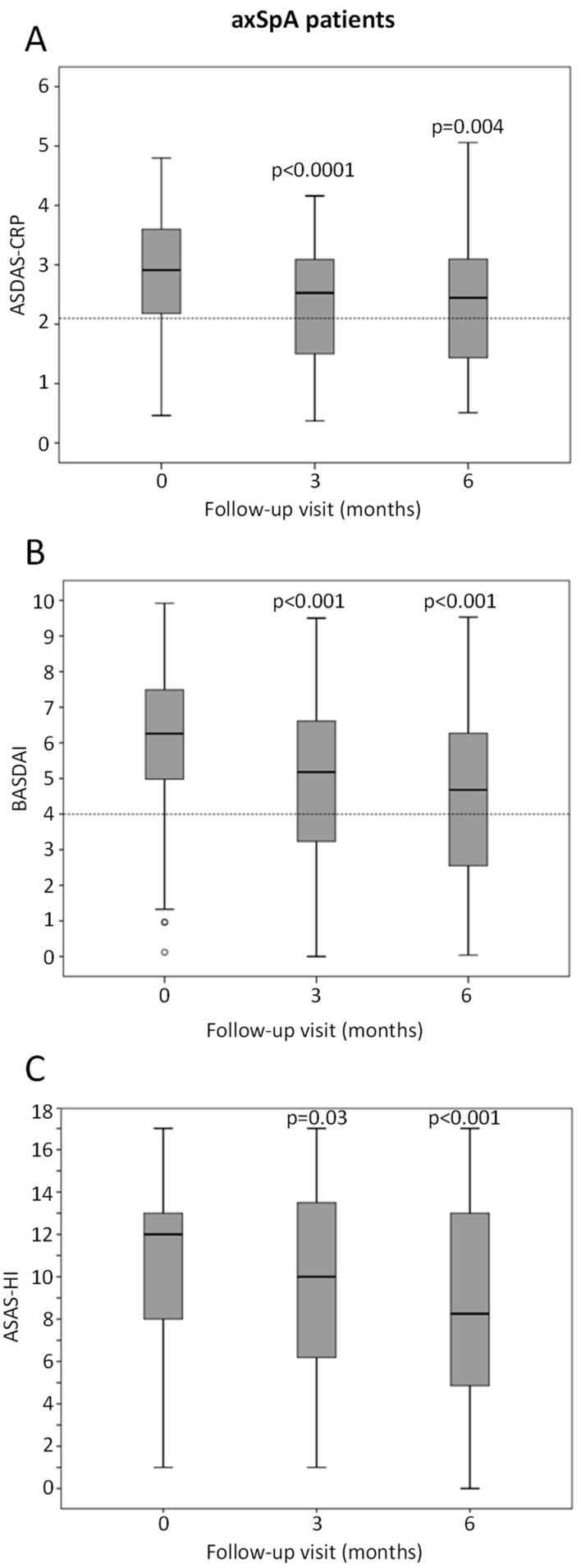
Box and whiskers plots illustrating changes in ASDAS-CRP, BASDAI and ASAS-HI in axSpA patients at baseline, 3 and 6 months of treatment with GLM. Data are presented as median, 25th/75th percentiles and maximum/minimum recorded values. Small open dots represent outliers, and horizontal dotted lines represent (**A**) a cut-off value of 2.1 (<2.1 defined as low disease activity according to ASDAS-CRP), (**B**) a cut-off value of 4 (BASDAI score ≥ 4 suggests suboptimal control of disease). Change in ASAS-HI in axSpA patients are also shown in (**C**). Statistically significant differences compared to baseline values are represented by *p*-values.

In axSpA, after 6 months of GLM treatment, 32.5% (95% CI: 18.6–49.1%) patients achieved at least LDA and 22.5% (95% CI: 10.8–38.5%) achieved remission according to ASDAS-CRP. The percentage of patients achieving a 50% improvement in BASDAI (BASDAI 50) was 16.0% (95% CI: 7.2–29.1%). Median ASDAS-CRP score decreased from 2.91 (IQR: 2.18–3.6) at baseline to 2.45 (IQR: 1.44–3.09; *p* = 0.004) at 6 months (Figure 2A). A similar decrease was observed for BASDAI; 6.26 (IQR: 4.92–7.53) at baseline to 4.68 (IQR: 2.55–6.27; *p* < 0.001) at 6 months (Figure 2B) and for ASAS-HI; 12 (IQR: 8–13) at baseline to 8.25 (IQR: 4.86–13; *p* < 0.001) at 6 months (Figure 2C). A mean decrease in ESR (approximately two-fold reduction) and PGA (about a 20% decrease) was also observed at 6 months following GLM treatment (Table 4).

### 3.6. Effect of Concomitant Methotrexate Treatment in PsA and AxSpA Patients

Sub-analysis was also performed to evaluate the efficacy of GLM on the primary efficacy outcomes in PsA and axSpA patients with and without MTX at 6 months. In PsA patients, we observed that MDA was achieved in 22.6% (7 out of 31) patients with MTX compared to 39% (16 out of 41) patients without MTX; this difference did not achieve statistical significance (*p* = 0.14). In axSpA patients, we observed that LDA (according to ASDAS- CRP) was achieved in 22.2% (2 out of 9) patients with MTX compared to 35.5% (11 out of 31) patients without MTX; this difference did not achieve statistical significance (*p* = 0.69). This sub-analysis suggests that concomitant MTX did not influence the efficacy of GLM in patients with PsA and axSpA in our cohort.

### 3.7. QoL Assessment Using the EQ-5D-5L

QoL scores decreased (i.e., improvement) across each of the five domains of the EQ-5D-5L from baseline to 6 months (Table 5). For mobility, a decrease of 13.8% (*p* = 0.001) was observed, 9.9% for self-care, 15.4% (*p* < 0.001) for usual activities, 6.8% (*p* = 0.012) for pain/discomfort and a reduction of 9.0% for anxiety/depression. The mean score of patients’ “health today” increased (representing an improvement) from 52.5 ± 21.3 at baseline to 58.5 ± 20.4 (*p* = 0.009) at 6 months. The mean EQ-5D-5L index also increased from 0.72 ± 0.15 at baseline to 0.78 ± 0.12 (*p* < 0.001) at 6 months and a non-significant decrease in EQ-VAS from 54 ± 22 at baseline to 52.7 ± 24.5 was observed (*p* = 0.57). Similar trends in improvement were also seen when stratifying patients by diagnosis (RA, PsA, axSpA) (Table 5).

### 3.8. Reasons for Discontinuation

During the 6-month treatment period, a total of 29 patients (14.9%) interrupted the study; of whom 19 (9.8%) discontinued due to a definitive interruption of GLM treatment, 8 were lost to follow-up, 1 interrupted due to lack of compliance and 1 for an unspecified reason. The mean time when interruption occurred was 1.96 ± 1.7 months after commencing GLM treatment. Of the 19 GLM definitive treatment interruptions, the majority were due to an issue with efficacy, with 11 (5.7%) reporting as lack of therapeutic effect and 3 (1.5%) reporting as loss of efficacy. Four treatment interruptions were due to adverse events, with two (1%) of these AEs considered related to GLM (both events occurring in patients with PsA) and two (1%) considered unrelated to GLM (both events occurring in patients with axSpA). The remaining treatment interruption was due to an unspecified other reason (Table 6). Overall, patients with RA had the highest proportion of study interruptions; however, none of the discontinuations due to AEs occurred in these patients.

## 4. Discussion

The main findings from the present real-life prospective study in Italy confirm the effectiveness of GLM as a second-line anti-TNFα for the treatment of RA, PsA and axSpA. In this analysis, with up to 6 months of treatment, GLM was found to be effective in achieving primary outcome measures to a similar extent in patients with RA, PsA and axSpA.

This real-life prospective study specifically focused on the effectiveness of GLM in a subgroup of patients who are not normally included in clinical trials, and our findings reveal that in patients with spondyloarthropathies (axSpA and PsA) as well as RA, who need to discontinue their first anti-TNF drug, the decision to switch to GLM can be considered a suitable therapeutic choice. These findings are in agreement with results reported in other recent real-life studies [21,22,23,24,25,26].

While several observational studies have focused on the long-term drug survival of GLM given as a second-line treatment in inflammatory arthritis, few studies have specifically evaluated the effectiveness of GLM in this setting. In a retrospective longitudinal study performed in Spain by Alegre-Sancho and colleagues, the long-term effectiveness and persistence of GLM as a second biological drug in patients with PsA (N = 131) and axSpA (N = 79) were evaluated [21]. In PsA patients, DAS28 decreased from 4.0 ± 1.3 at baseline to 2.5 ± 1.2 at 3 months. In axSpA patients, BASDAI score at baseline was 5.5 ± 2.1 and decreased to 3.9 ± 2.0 at 3 months. In our cohort, which shares similar clinical characteristics with the Spanish study in terms of disease duration, comorbidities and concomitant medication, we observed a similar reduction in DAS28-CRP by 3 months in PsA patients (3.8 ± 0.99 at baseline to 2.8 ± 1.1; *p* < 0.0001) as well as a decrease in BASDAI in axSpA patients (6.02 ± 2.09 at baseline to 4.9 ± 2.2; *p* < 0.001), although baseline disease activity was higher in our axSpA population. Patients with RA were not included in the study by Alegre-Sancho et al.; however, a similar study (in terms of design and patient characteristics) undertaken in Turkey (GO-BEYOND Turkey) did include RA patients [22]. Akar et al. evaluated persistence and changes in disease activity measures over 2 years in 60 RA and 269 axSpA patients after GLM treatment. Although the majority of patients in this study were biologic naïve, their analysis did stratify a subset of patients who were biologic experienced. In this subgroup of the RA population (N = 7), DAS28-CRP decreased from 4.8 to 3.0 after 6 months, similar to what we have observed in our larger cohort in Italy (N = 39; from 4.1 at baseline to 2.8 at 6 months; *p* < 0.001). In patients with axSpA previously treated with an anti-TNF drug (N = 28), a two-fold reduction was seen for ASDAS and BASDAI after 6 months, a greater improvement than what we have observed. However, patients with axSpA were considerably younger (40 vs. 51 years) and presented with lower CRP levels, ASDAS and BASDAI scores at baseline.

In the recent real-life retrospective analysis of the Italian GISEA registry, the effectiveness and persistence of GLM in biologic inadequate responder (IR) patients with RA, PsA and axSpA were investigated [26]. Disease activity in the subgroup of RA patients who were IR to 1 biologic (N = 94) improved after 6 months, as seen by a marked reduction in DAS28 from 4.9 ± 1.2 at baseline to 3.7 ± 1.1 at 6 months. These patients had a higher burden of disease activity at baseline compared to patients included in our study (DAS28 of 4.9 vs. 3.7), but the absolute decrease (1.2 units) was identical to what we observed. A good EULAR response was achieved in 52% of RA patients who had previously failed 1 biologic compared to 61.9% in our cohort. In the GISEA registry study, among PsA patients with axial involvement previously failing 1 biologic, 41% had BASDAI < 4 at 6 months. In patients with axSpA, the percentage who achieved BASDAI 50 at 6 months was 40%, while approximately 60% of all patients with SpA achieved LDA, and 23% were in remission according to ASDAS. Compared to GISEA, our findings are favorable in terms of improvement in disease activity and the clinical response observed in patients with RA and PsA; however, clinical outcomes in patients with axSpA as measured by BASDAI 50 and LDA were less favorable. In our study, patients with axSpA tended to be older (mean 51 vs. 46 years) and presented with a higher burden of comorbidities (76.6% vs. 41%), which may, in part, explain these observed differences.

The post hoc analysis of the prospective GO-NICE study undertaken in Germany also evaluated the effectiveness of GLM by line of treatment in patients with RA, PsA and AS [25]. In patients with RA (N = 104) given GLM as a second-line biologic, DAS-28 decreased from 4.9 ± 1.3 to 3.4 ± 1.5 and 19.5% were in remission at 6 months. The Psoriatic Arthritis Response Criteria [42] in patients with PsA showed a 51.2% improvement at 6 months and BASDAI score decreased from 4.9 ± 2 to 3.3 ± 2.2 in AS patients. Similar results were also seen in the prospective post hoc analysis of the prospective GO-PRACTICE study performed in France in second-line biologic patients with axSpA, where BASDAI was observed to decrease to a similar extent (from 5.7 to 3.9) at 6 months [24]. Results from GO-NICE and GO-PRACTICE corroborate with our findings up to 6 months in terms of DAS-28 improvement in patients with RA (approximately 30% reduction vs. baseline). A similar improvement was also seen in the axSpA group for BASDAI despite the fact that our cohort included slightly older patients (51 ± 13.2 years vs. 45.3 ± 12.3 years and 46.8 ± 12.3 years for GO-NICE and GO-PRACTICE, respectively).

Collectively, results from the present study, in conjunction with these real-life studies, provide evidence on the effectiveness of GLM as a second-line biologic in patients with RA, PsA and axSpA for up to 6 months.

## 5. Study Limitations and Strengths

The main limitation of this study is intrinsic to its observational design, thus potentially affecting the results of the analysis.

Strengths of this study include the prospective design, with the accuracy of data collection according to systematic protocol-specified visits at baseline, 3 and 6 months and the enrolment of a very selected study population including only patients with RA, PsA or axSpA who were switched to GLM after previous failure with another anti-TNFα.

## 6. Conclusions

Considering the high rate of failure following first-line anti-TNF treatments, rheumatologists are frequently faced with the need to consider a switch in anti-TNF as second-line therapy. In this respect, the availability of real-life data such as those in the present study provide useful information to guide therapeutic decisions. In this large real-life prospective cohort, the use of GLM after first-line anti-TNF failure was found to be an effective and valuable option in patients with RA, PsA and axSpA.

## Figures and Tables

**Table 1:** Baseline characteristics of RA, PsA and axSpA patients.

Characteristics	All Patients *n* = 194	RA *n* = 39	PsA *n* = 91	AxSpA *n* = 64
Female gender (%)	110 (56.7)	29 (74.4)	47 (51.6)	34 (53.1)
Age (years)	53.2 ± 12	55.4 ± 11.4	53.7 ± 11.3	51 ± 13.2
BMI (kg/m^2^)	26.5 ± 5.1	25.6 ± 6.5	27.2 ± 5	25.9 ± 4.1
Disease duration (years)	9.9 ± 8.3	11 ± 9.1	9.8 ± 7.8	9.2 ± 8.5
Current smoker	36 (18.6)	9 (23.1)	16 (17.6)	11 (17.2)
ACPA+, *n* (%)	-	17 (43.6)	-	-
RF+, *n* (%)	-	18 (46.2)	-	-
HLAB27+, *n* (%)	-	-	-	18 (28.1)
Tender joint count (0–28)	-	6.7 ± 4.9	5.7 ± 4.6	-
Swollen joint count (0–28)	-	2.3 ± 2.3	2.1 ± 3.4	-
PGA	62.9 ± 21.7	63.7 ± 21	60.9 ± 20.9	65.2 ± 23.2
PASI	-	-	2.9 ± 9.7	-
CRP (mg/L)	6 ± 10.9	10.2 ± 18.1	4.7 ± 7.7	5.4 ± 8
ESR (mm/h)	21.4 ± 21.8	23.6 ± 22.1	20.8 ± 21.6	20.9 ± 22.3
DAS28-CRP	-	4.1 ± 0.94	3.8 ± 0.99	
ASDAS-CRP	-	-	-	2.9 ± 0.97
BASDAI	-	-	-	6 ± 2.1
ASAS-HI	-	-	-	10.6 ± 3.9
Extra-articular manifestations, (%)				
Skin psoriasis	-	-	40 (44)	8 (12.5)
Enthesitis	-	-	30 (33)	10 (15.6)
Nail psoriasis	-	-	17 (18.7)	1 (1.6)
Dactylitis	-	-	4 (4.4)	2 (3.1)
Comorbidities, *n* (%)	135 (69.6)	26 (66.7)	60 (65.9)	49 (76.6)
Hypertension	60 (30.9)	12 (30.8)	28 (30.8)	20 (31.3)
Thyroid disease	27 (13.9)	6 (15.4)	14 (15.4)	7 (10.9)
Dyslipidemia	26 (13.4)	6 (15.4)	11 (12.1)	9 (14.1)
Other diseases	70 (36.1)	12 (30.8)	28 (30.8)	30 (46.9)

ACPA—anti-citrullinated protein antibody; ASAS-HI—Assessment of SpondyloArthritis international society Health Index; ASDAS-CRP—ankylosing spondylitis disease activity score with CRP; axSpA—axial spondyloarthritis; BASDAI—Bath Ankylosing Spondylitis Disease Activity Index; BMI—body mass index; CRP—C-reactive protein; DAS28—Disease Activity Score 28; ESR—erythrocyte sedimentation rate; HLAB27—human leukocyte antigen B27; PASI—psoriasis area and severity index; PGA—patient global assessment; PsA—psoriatic arthritis; RA—rheumatoid arthritis; RF—rheumatoid factor. Data are reported as mean ± standard deviation or frequencies (number and %).

**Table 2:** Concomitant medication in RA, PsA and axSpA patients at baseline.

Characteristics	All Patients *n* = 194	RA *n* = 39	PsA *n* = 91	AxSpA *n* = 64
Concomitant medication	142 (73.2)	39 (100)	60 (65.9)	43 (67.2)
Methotrexate	85 (43.8)	37 (94.9)	33 (36.3)	15 (23.4)
NSAIDs	45 (23.2)	5 (12.8)	21 (23.1)	19 (29.7)
Corticosteroids	44 (22.7)	15 (38.5)	16 (17.6)	13 (20.3)
Sulfasalazine	13 (6.7)	3 (7.7)	3 (3.3)	7 (10.9)
Previous anti-TNFα, n (%)				
Etanercept	86 (44.3)	21 (53.8)	48 (52.7)	17 (26.6)
Adalimumab	82 (42.3)	9 (23.1)	38 (41.8)	35 (54.7)
Infliximab	17 (8.8)	5 (12.8)	1 (1.1)	11 (17.2)
Certolizumab	9 (4.6)	4 (10.3)	4 (4.4)	1 (1.6)
Duration of previous anti-TNFα (months)				
Etanercept	53.3 ± 48.9	37.8 ± 42.5	53.8 ± 48.5	72.1 ± 53.3
Adalimumab	42.7 ± 35.8	62.2 ± 41	51.5 ± 37.7	28.2 ± 26.6
Infliximab	76.9 ± 63.4	75.4 ± 45.4	6 ± 0	84.1 ± 71.1
Certolizumab	30.4 ± 16.7	34.3 ± 20.2	28.8 ± 17	22 ± 0

NSAID—non-steroidal anti-inflammatory drugs; TNFα—tumor necrosis alpha; axSpA—axial spondyloarthritis; PsA—psoriatic arthritis; RA—rheumatoid arthritis. Data are reported as mean ± standard deviation or frequencies (number and %).

**Table 3:** Reasons for switching from anti-TNFα inhibitor to golimumab.

	All Patients				
Reason	Adalimumab	Certolizumab	Etanercept	Infliximab	Total, *n* (%)
Loss of efficacy	67 (81.7)	9 (100)	72 (83.7)	7 (41.2)	155 (79.9)
Injection site or infusion reaction	2 (2.4)	0 (0)	6 (7)	3 (17.6)	11 (5.7)
Other adverse events	11 (13.4)	0 (0)	6 (7)	5 (29.4)	22 (11.3)
Lack of compliance	1 (1.2)	0 (0)	1 (1.2)	2 (11.8)	4 (2.1)
Patient dissatisfaction	1 (1.2)	0 (0)	1 (1.2)	0 (0)	2 (1)
	**RA patients**				
Loss of efficacy	8 (88.9)	4 (100)	15 (71.4)	4 (80)	31 (79.5)
Injection site or infusion reaction	0 (0)	0 (0)	3 (14.3)	0 (0)	3 (7.7)
Other adverse events	1 (11.1)	0 (0)	2 (9.5)	0 (0)	3 (7.7)
Lack of compliance	0 (0)	0 (0)	1 (4.8)	1 (20)	2 (5.1)
Patient dissatisfaction	0 (0)	0 (0)	0 (0)	0 (0)	0 (0)
	**PsA patients**				
Loss of efficacy	31 (81.6)	4 (100)	41 (85.4)	0 (0)	76 (83.5)
Injection site or infusion reaction	2 (5.3)	0 (0)	3 (6.3)	1 (100)	6 (6.6)
Other adverse events	5 (13.2)	0 (0)	4 (8.3)	0 (0)	9 (9.9)
Lack of compliance	0 (0)	0 (0)	0 (0)	0 (0)	0 (0)
Patient dissatisfaction	0 (0)	0 (0)	0 (0)	0 (0)	0 (0)
	**axSpA patients**				
Loss of efficacy	28 (80.0)	1 (100)	16 (94.1)	3 (27.3)	48 (75.0)
Injection site or infusion reaction	0 (0)	0 (0)	0 (0)	2 (18.2)	2 (3.1)
Other adverse events	5 (14.3)	0 (0)	0 (0)	5 (45.5)	10 (15.6)
Lack of compliance	1 (2.9)	0 (0)	0 (0)	1 (9.1)	2 (3.1)
Patient dissatisfaction	1 (2.9)	0 (0)	1 (5.9)	0 (0)	2 (3.1)

AxSpA—axial spondyloarthritis; PsA—psoriatic arthritis; RA—rheumatoid arthritis. Data presented as numbers and %.

**Table 4:** Changes in disease activity measures after golimumab treatment in RA, PsA and axSpA patients.

	RA Patients			PsA Patients			axSpA Patients		
	Baseline (*n* = 39)	6 Months (*n* = 29)	*p*-Value	Baseline (*n* = 91)	6 Months (*n* = 79)	*p*-Value	Baseline (*n* = 64)	6 Months (*n* = 54)	*p*-Value
CRP (mg/L)	10.2 ± 18.1	5.8 ± 7.6	0.83	4.7 ± 7.7	3.7 ± 5.2	0.78	5.4 ± 8	4.95 ± 10.8	0.36
ESR (mm/h)	23.6 ± 22.1	25.3 ± 24.8	0.61	20.8 ± 21.6	17.95 ± 16	0.68	20.9 ± 22.3	12.4 ± 12.9	0.18
SJC (28 joints)	2.3 ± 2.3	0.62 ± 1.5	**<0.001**	2.1 ± 3.4	0.55 ± 1.3	**<0.001**	-	-	-
TJC (28 joints)	6.7 ± 4.9	2.4 ± 3	**<0.001**	5.7 ± 4.6	2.6 ± 4	**<0.001**	-	-	-
PASI	-	-	**-**	2.9 ± 9.7	0.94 ± 3.8	0.11	-	-	-
PGA	63.7 ± 21	38.1 ± 21.1	**<0.001**	60.9 ± 20.9	42.1 ± 25	**<0.001**	65.2 ± 23.2	51.1 ± 26.8	**0.001**

AxSpA—axial spondyloarthritis; CRP—C-reactive protein; ESR—erythrocyte sedimentation rate; PASI—psoriasis area and severity index; PGA—patient global assessment; PsA—psoriatic arthritis; RA—rheumatoid arthritis; RF—rheumatoid factor; SJC—swollen joint count based on 28 joints; TJC—tender joint count based on 28 joints. Statistically significant differences compared to baseline values are represented by *p*-values in bold text. Data are presented as mean ± standard deviation or frequencies (number and %).

**Table 5:** Evaluation of QoL by EQ-5D-5L in RA, PsA and axSpA patients.

	All Patients			RA Patients			PsA Patients			axSpA Patients		
Domain (Slight Problem vs. No Problem)	Baseline (N = 188)	6 Months (N = 146)	*p*-Value	Baseline (N = 39)	6 Months (N = 27)	*p*-Value	Baseline (N = 88)	6 Months (N = 70)	*p*-Value	Baseline (N = 61)	6 Months (N = 49)	*p*-Value
1. Mobility	152 (80.9)	98 (67.1)	**0.001**	29 (74.4)	17 (63)	0.51	71 (80.7)	49 (70)	**0.02**	52 (85.2)	32 (65.3)	**0.049**
2. Self-care	128 (68.1)	85 (58.2)	0.08	26 (66.7)	17 (63)	1	58 (65.9)	43 (61.4)	0.7	44 (72.1)	25 (51)	**0.049**
3. Usual activities	168 (89.4)	108 (74.0)	**<0.001**	33 (84.6)	21 (77.8)	1	79 (89.8)	50 (71.4)	**0.002**	56 (91.8)	37 (75.5)	**0.016**
4. Pain/discomfort	184 (97.9)	133 (91.1)	**0.012**	38 (97.4)	25 (92.6)	1	86 (97.7)	61 (87.1)	**0.016**	60 (98.4)	47 (95.9)	1
5. Anxiety/depression	125 (66.5)	84 (57.5)	0.19	25 (64.1)	15 (55.6)	0.73	57 (64.8)	40 (57.1)	0.44	43 (70.5)	29 (59.2)	0.55
Health today	52.5 ± 21.3	58.5 ± 20.4	**0.009**	56.8 ± 18.5	59.3 ± 17.3	1	54.9 ± 20	60.5 ± 19.9	**0.013**	46.3 ± 23.6	55 ± 22.6	0.17
EQ-5D-5L index	0.72 ± 0.15	0.78 ± 0.12	**<0.001**	0.71 ± 0.19	0.78 ± 0.13	0.08	0.73 ± 0.11	0.79 ± 0.12	**0.004**	0.69 ± 0.17	0.77 ± 0.13	**0.001**
EQ-VAS	54 ± 22	52.7 ± 24.5	0.57	61 ± 19	53.2 ± 20.6	0.07	54.9 ± 21.4	53.4 ± 24.9	0.78	47.7 ± 23.5	51.4 ± 26.4	0.72

AxSpA—axial spondyloarthritis; EQ-5D-5L index—EuroQoL 5-Dimension 5-Level (index); EQ-VAS—EuroQol Visual Analogue Scale; PsA—psoriatic arthritis; RA—rheumatoid arthritis. Statistically significant differences compared to baseline values are represented by *p*-values in bold text. Data presented as frequency and %.

**Table 6:** Reasons for golimumab interruption up to 6 months.

Reason	All Patients *n* = 194	RA *n* = 39	PsA *n* = 91	AxSpA *n* = 64
Study interruption	29 (14.9)	9 (23.1)	10 (11)	10 (15.6)
Reason for study interruption				
Definitive interruption of GLM	19 (9.8)	5 (12.8)	5 (5.5)	9 (14.1)
Lost to follow-up	8 (4.1)	2 (5.1)	5 (5.5)	1 (1.6)
Lack of compliance	1 (0.52)	1 (2.6)	0 (0)	0 (0)
Other	1 (0.52)	1 (2.6)	0 (0)	0 (0)
Reason for GLM interruption				
Lack of therapeutic effect	11 (5.7)	3 (7.7)	2 (2.2)	6 (9.4)
Loss of efficacy	3 (1.5)	2 (5.1)	0 (0)	1 (1.6)
AEs related to GLM *	2 (1)	0 (0)	2 (2.2)	0 (0)
AEs not related to GLM **	2 (1)	0 (0)	0 (0)	2 (3.1)
Other	1 (0.52)	0 (0)	1 (1.1)	0 (0)

AE—adverse events; axSpA—axial spondyloarthritis; GLM—golimumab; PsA—psoriatic arthritis; RA—rheumatoid arthritis. * AEs related to GLM included eczema for one patient and epistaxis and erythema for the second patient. ** AEs not related to GLM were lung cancer and nosocomial infection.

## Data Availability

Data are available upon reasonable request. All data relevant to the study are included in the article. The datasets generated during or analyzed during the current study are not publicly available. All data used in this study were anonymized to respect the privacy of patients in line with applicable laws and regulations.

## References

[1] Feldmann M., Maini R.N. (2001). Anti-TNF Alpha Therapy of Rheumatoid Arthritis: What Have We Learned?. Annu. Rev. Immunol..

[2] Taylor P.C. (2001). Anti-TNF Therapy for Rheumatoid Arthritis and Other Inflammatory Diseases. Mol. Biotechnol..

[3] Palazzi C., D’Angelo S., Gilio M., Leccese P., Padula A., Olivieri I. (2015). Pharmacological Therapy of Spondyloarthritis. Expert Opin. Pharmacother..

[4] D’Angelo S., Malavolta N., Scambi C., Salvarani C., Caso F., Tirri E., Ramonda R., Quarta L., Erre G.L., Riva M. (2021). Quality of Life and Therapeutic Management of Axial Spondyloarthritis Patients in Italy: A 12-Month Prospective Observational Study. Clin. Exp. Rheumatol..

[5] van der Heijde D., Ramiro S., Landewé R., Baraliakos X., Van den Bosch F., Sepriano A., Regel A., Ciurea A., Dagfinrud H., Dougados M. (2017). 2016 Update of the ASAS-EULAR Management Recommendations for Axial Spondyloarthritis. Ann. Rheum. Dis..

[6] Gossec L., Baraliakos X., Kerschbaumer A., de Wit M., McInnes I., Dougados M., Primdahl J., McGonagle D.G., Aletaha D., Balanescu A. (2020). EULAR Recommendations for the Management of Psoriatic Arthritis with Pharmacological Therapies: 2019 Update. Ann. Rheum. Dis..

[7] Roda G., Jharap B., Neeraj N., Colombel J.-F. (2016). Loss of Response to Anti-TNFs: Definition, Epidemiology, and Management. Clin. Transl. Gastroenterol..

[8] Favalli E.G., Pregnolato F., Biggioggero M., Becciolini A., Penatti A.E., Marchesoni A., Meroni P.L. (2016). Twelve-Year Retention Rate of First-Line Tumor Necrosis Factor Inhibitors in Rheumatoid Arthritis: Real-Life Data From a Local Registry. Arthritis Care Res..

[9] Papagoras C., Voulgari P.V., Drosos A.A. (2010). Strategies after the Failure of the First Anti-Tumor Necrosis Factor Alpha Agent in Rheumatoid Arthritis. Autoimmun. Rev..

[10] Smolen J.S., Landewé R.B.M., Bijlsma J.W.J., Burmester G.R., Dougados M., Kerschbaumer A., McInnes I.B., Sepriano A., van Vollenhoven R.F., de Wit M. (2020). EULAR Recommendations for the Management of Rheumatoid Arthritis with Synthetic and Biological Disease-Modifying Antirheumatic Drugs: 2019 Update. Ann. Rheum. Dis..

[11] Keystone E.C., Genovese M.C., Hall S., Miranda P.C., Bae S.-C., Palmer W., Wu Z., Xu S., Hsia E.C. (2013). Golimumab in Patients with Active Rheumatoid Arthritis despite Methotrexate Therapy: Results through 2 Years of the GO-FORWARD Study Extension. J. Rheumatol..

[12] Kavanaugh A., McInnes I., Mease P., Krueger G.G., Gladman D., Gomez-Reino J., Papp K., Zrubek J., Mudivarthy S., Mack M. (2009). Golimumab, a New Human Tumor Necrosis Factor Alpha Antibody, Administered Every Four Weeks as a Subcutaneous Injection in Psoriatic Arthritis: Twenty-Four-Week Efficacy and Safety Results of a Randomized, Placebo-Controlled Study. Arthritis Rheum..

[13] Inman R.D., Davis J.C., van der Heijde D., Diekman L., Sieper J., Kim S.I., Mack M., Han J., Visvanathan S., Xu Z. (2008). Efficacy and Safety of Golimumab in Patients with Ankylosing Spondylitis: Results of a Randomized, Double-Blind, Placebo-Controlled, Phase III Trial. Arthritis Rheum..

[14] Sieper J., van der Heijde D., Dougados M., Maksymowych W.P., Scott B.B., Boice J.A., Berd Y., Bergman G., Curtis S., Tzontcheva A. (2015). A Randomized, Double-Blind, Placebo-Controlled, Sixteen-Week Study of Subcutaneous Golimumab in Patients with Active Nonradiographic Axial Spondyloarthritis. Arthritis Rheumatol..

[15] Palazzi C., D’angelo S., Gilio M., Leccese P., Padula A., Olivieri I. (2017). Golimumab for the Treatment of Axial Spondyloarthritis. Expert Opin. Biol. Ther..

[16] Tahir Z., Kavanaugh A. (2018). The Role of Golimumab in Inflammatory Arthritis. A Review of the Evidence. Ther. Adv. Musculoskelet. Dis..

[17] Smolen J.S., Kay J., Doyle M.K., Landewé R., Matteson E.L., Wollenhaupt J., Gaylis N., Murphy F.T., Neal J.S., Zhou Y. (2009). Golimumab in Patients with Active Rheumatoid Arthritis after Treatment with Tumour Necrosis Factor Alpha Inhibitors (GO-AFTER Study): A Multicentre, Randomised, Double-Blind, Placebo-Controlled, Phase III Trial. Lancet.

[18] Smolen J.S., Kay J., Doyle M., Landewé R., Matteson E.L., Gaylis N., Wollenhaupt J., Murphy F.T., Xu S., Zhou Y. (2015). Golimumab in Patients with Active Rheumatoid Arthritis after Treatment with Tumor Necrosis Factor α Inhibitors: Findings with up to Five Years of Treatment in the Multicenter, Randomized, Double-Blind, Placebo-Controlled, Phase 3 GO-AFTER Study. Arthritis Res. Ther..

[19] Iannone F., Santo L., Anelli M.G., Bucci R., Semeraro A., Quarta L., D’Onofrio F., Marsico A., Carlino G., Casilli O. (2017). Golimumab in Real-Life Settings: 2 Years Drug Survival and Predictors of Clinical Outcomes in Rheumatoid Arthritis, Spondyloarthritis, and Psoriatic Arthritis. Semin. Arthritis Rheum..

[20] Michelsen B., Sexton J., Wierød A., Bakland G., Rødevand E., Krøll F., Kvien T.K. (2020). Four-Year Follow-up of Inflammatory Arthropathy Patients Treated with Golimumab: Data from the Observational Multicentre NOR-DMARD Study. Semin. Arthritis Rheum..

[21] Alegre-Sancho J.J., Juanola X., Rodríguez-Heredia J.M., Manero J., Villa-Blanco I., Laiz A., Arteaga M.J., Cea-Calvo L., González C.M. (2021). Effectiveness and Persistence of Golimumab as a Second Biological Drug in Patients with Spondyloarthritis: A Retrospective Study. Medicine.

[22] Akar S., Kalyoncu U., Dalkilic E., Emmungil H., Aziz A., Esen Y., Koc T. (2021). GO-BEYOND: A Real-World Study of Persistence of Golimumab in Patients with Axial Spondyloarthritis and Rheumatoid Arthritis in Turkey. Immunotherapy.

[23] Flipo R.-M., Tubach F., Goupille P., Lespessailles E., Harid N., Sequeira S., Bertin P., Fautrel B. (2021). Real-Life Persistence of Golimumab in Patients with Chronic Inflammatory Rheumatic Diseases: Results of the 2-Year Observational GO-PRACTICE Study. Clin. Exp. Rheumatol..

[24] Goupille P., Bertin P., Tubach F., Lespessailles E., Harid N., Sequeira S., Fayette J.-M., Fautrel B., Flipo R.-M. (2022). Real-Life Golimumab Persitence in Patients with Axial Spondyloarthritis: Post-Hoc Results of the Prospective Observational Cohort Study, GO-PRACTICE. Clin. Exp. Rheumatol..

[25] Krüger K., Burmester G.R., Wassenberg S., Thomas M.H. (2020). Golimumab as the First-, Second-, or at Least Third-Line Biologic Agent in Patients with Rheumatoid Arthritis, Psoriatic Arthritis, or Ankylosing Spondylitis: Post Hoc Analysis of a Noninterventional Study in Germany. Rheumatol. Ther..

[26] Iannone F., Favalli E.G., Caporali R., D’Angelo S., Cantatore F.P., Sarzi-Puttini P., Foti R., Conti F., Carletto A., Gremese E. (2021). Golimumab Effectiveness in Biologic Inadequate Responding Patients with Rheumatoid Arthritis, Psoriatic Arthritis and Spondyloarthritis in Real-Life from the Italian Registry GISEA. Joint Bone Spine.

[27] Aletaha D., Neogi T., Silman A.J., Funovits J., Felson D.T., Bingham C.O., Birnbaum N.S., Burmester G.R., Bykerk V.P., Cohen M.D. (2010). 2010 Rheumatoid Arthritis Classification Criteria: An American College of Rheumatology/European League Against Rheumatism Collaborative Initiative. Ann. Rheum. Dis..

[28] Taylor W., Gladman D., Helliwell P., Marchesoni A., Mease P., Mielants H., CASPAR Study Group (2006). Classification Criteria for Psoriatic Arthritis: Development of New Criteria from a Large International Study. Arthritis Rheum..

[29] Rudwaleit M., van der Heijde D., Landewé R., Listing J., Akkoc N., Brandt J., Braun J., Chou C.T., Collantes-Estevez E., Dougados M. (2009). The Development of Assessment of SpondyloArthritis International Society Classification Criteria for Axial Spondyloarthritis (Part II): Validation and Final Selection. Ann. Rheum. Dis..

[30] European Medicines Agency Simponi—Summary of Product Characteristics. https://www.ema.europa.eu/en/medicines/human/EPAR/simponi.

[31] van Riel P.L.C.M. (2014). The Development of the Disease Activity Score (DAS) and the Disease Activity Score Using 28 Joint Counts (DAS28). Clin. Exp. Rheumatol..

[32] Lukas C., Landewé R., Sieper J., Dougados M., Davis J., Braun J., van der Linden S., van der Heijde D., Assessment of SpondyloArthritis international Society (2009). Development of an ASAS-Endorsed Disease Activity Score (ASDAS) in Patients with Ankylosing Spondylitis. Ann. Rheum. Dis..

[33] Machado P., Landewé R., Lie E., Kvien T.K., Braun J., Baker D., van der Heijde D., Assessment of SpondyloArthritis International Society (2011). Ankylosing Spondylitis Disease Activity Score (ASDAS): Defining Cut-off Values for Disease Activity States and Improvement Scores. Ann. Rheum. Dis..

[34] Garrett S., Jenkinson T., Kennedy L.G., Whitelock H., Gaisford P., Calin A. (1994). A New Approach to Defining Disease Status in Ankylosing Spondylitis: The Bath Ankylosing Spondylitis Disease Activity Index. J. Rheumatol..

[35] Kiltz U., van der Heijde D., Boonen A., Cieza A., Stucki G., Khan M.A., Maksymowych W.P., Marzo-Ortega H., Reveille J., Stebbings S. (2015). Development of a Health Index in Patients with Ankylosing Spondylitis (ASAS HI): Final Result of a Global Initiative Based on the ICF Guided by ASAS. Ann. Rheum. Dis..

[36] EuroQol Group (1990). EuroQol—A New Facility for the Measurement of Health-Related Quality of Life. Health Policy.

[37] Smolen J.S., Landewé R., Bijlsma J., Burmester G., Chatzidionysiou K., Dougados M., Nam J., Ramiro S., Voshaar M., van Vollenhoven R. (2017). EULAR Recommendations for the Management of Rheumatoid Arthritis with Synthetic and Biological Disease-Modifying Antirheumatic Drugs: 2016 Update. Ann. Rheum. Dis..

[38] Coates L.C., Fransen J., Helliwell P.S. (2010). Defining Minimal Disease Activity in Psoriatic Arthritis: A Proposed Objective Target for Treatment. Ann. Rheum. Dis..

[39] Fransen J., van Riel P.L.C.M. (2005). The Disease Activity Score and the EULAR Response Criteria. Clin. Exp. Rheumatol..

[40] Carmona L., Gómez-Reino J.J. (2006). Survival of TNF Antagonists in Spondylarthritis Is Better than in Rheumatoid Arthritis. Data from the Spanish Registry BIOBADASER. Arthritis Res. Ther..

[41] Clopper C.J., Pearson E.S. (1934). The Use of Confidence or Fiducial Limits Illustrated in the Case of the Binomial. Biometrika.

[42] Mease P.J. (2011). Measures of Psoriatic Arthritis: Tender and Swollen Joint Assessment, Psoriasis Area and Severity Index (PASI), Nail Psoriasis Severity Index (NAPSI), Modified Nail Psoriasis Severity Index (MNAPSI), Mander/Newcastle Enthesitis Index (MEI), Leeds Enthesitis Index (LEI), Spondyloarthritis Research Consortium of Canada (SPARCC), Maastricht Ankylosing Spondylitis Enthesis Score (MASES), Leeds Dactylitis Index (LDI), Patient Global for Psoriatic Arthritis, Dermatology Life Quality Index (DLQI), Psoriatic Arthritis Quality of Life (PsAQOL), Functional Assessment of Chronic Illness Therapy-Fatigue (FACIT-F), Psoriatic Arthritis Response Criteria (PsARC), Psoriatic Arthritis Joint Activity Index (PsAJAI), Disease Activity in Psoriatic Arthritis (DAPSA), and Composite Psoriatic Disease Activity Index (CPDAI). Arthritis Care Res..

